# Coumaroyl Flavonol Glycosides and More in Marketed Green Teas: An Intrinsic Value beyond Much-Lauded Catechins

**DOI:** 10.3390/molecules25081765

**Published:** 2020-04-11

**Authors:** Lorenzo Candela, Marialuisa Formato, Giuseppina Crescente, Simona Piccolella, Severina Pacifico

**Affiliations:** Department of Environmental Biological and Pharmaceutical Sciences and Technologies, University of Campania “Luigi Vanvitelli”, Via Vivaldi 43, 81100 Caserta, Italy; lorenzocandela94@gmail.com (L.C.); marialuisa.formato@unicampania.it (M.F.); giuseppina.crescente@unicampania.it (G.C.); simona.piccolella@unicampania.it (S.P.)

**Keywords:** marketed green tea, ultrasound assisted maceration, UHPLC-HRMS metabolic profile, coumaroyl flavonol glycosides

## Abstract

Marketed green teas (GTs) can highly vary in their chemical composition, due to different origins, processing methods, and a lack of standardization of GT-based products. Consequently, biological activities become difficult to correlate to the presence/content of certain constituents. Herein, ultra-high-performance liquid chromatography (UHPLC) combined with high-resolution tandem mass spectrometry (HR MS/MS) was successfully applied to six commercial GT products, extracted by ethanol sonication, to disclose their polyphenol profile beyond the well-known catechins. The relative abundance of each class of metabolites was correlated to antiradical and antilipoperoxidant data through hierarchical clustering analysis, since it reasonably affects the beneficial properties of the product that reaches the consumer. The thiobarbituric acid reactive substances (TBARS) assay demonstrated that GT extracts effectively counteracted the UV-induced lipoperoxidation of hemp oil, which is highly rich in Polyunsaturated Fatty Acids (PUFAs), and therefore highly unstable. The Relative Antioxidant Capacity Index (RACI) comprehensively emphasized that gunpower and blend in filter GTs appeared to be the less active matrices, and except for a GT-based supplement, the Sencha GT, which was particularly rich in flavonol glycosides, was the most active, followed by Bancha GT.

## 1. Introduction

Tea from leaves of *Camellia sinensis* (Theaceae family) is one of the most popular beverages, whose consumption worldwide has a long history. It has always played a leading role in Chinese history and in China’s relations with near and far foreign cultures. It has long been considered a luxury product, highly sought after by all the people who acquired its stimulating taste [1]. The growing popularity of this beverage with valuable medicinal properties quickly spread to other East Asian cultures, especially Japan. The tea introduction in Europe defined its approval, fame, and its big business so much so that its global market was valued at nearly 50 billion U.S. dollars in 2017 and is expected to rise to over 73 billion dollars by 2024 [2].

The huge tea variety on the market is the result of different processing methods, in which withering, rolling, oxidation, and drying are the common steps [3]. Dark teas undergo high oxidation levels, whereas green tea completely skips oxidation, avoiding the browning process and thus preserving the green color of the leaf matrix and its richness in bioactive polyphenols. Catechins comprise 80–90% of flavonoid content [4], and they are especially pointed out as green tea (GT) catechins, as flavan-3-ols in GT mostly show a galloyl moiety on the C-3 carbon of catechin core or a pyrogallol B-ring. Catechins are nutraceutical compounds, exerting a preventive efficacy in offsetting oxidant species over-genesis in normal cells, and for their ability to halt or reverse oxidative stress-related diseases [5,6,7]. In this framework, multiple scientific evidences support the catechin-related health benefits of green tea consumption that include blood flow improvement, cardiovascular disease prevention, toxins elimination, and resistance enhancement to various diseases [8,9]. Cancer prevention with green tea was also recently reviewed; green tea, beyond the ability to delay cancer onset and to reduce its incidence, was reported as a useful strategy in secondary and tertiary cancer prevention [10]. Furthermore, cardiometabolic diseases, such as the ongoing metabolic syndrome, dyslipidemia, and diabetes, are reported to be beneficially alleviated by green tea [11]. These findings appeared to promote the therapeutic implementation of green tea [12]. Thus, in the great tea world, green tea has become very widespread over the last decades, and among the different Chinese and Japanese green tea leaves currently available on the market, also thanks to food sector operators and mass media advertisement, various supplements enriched in green tea catechins were prepared. Indeed, scientific data are uncertainly translated into meaningful benefits [13], which is mainly due to the instability and low bioavailability of green tea compounds. Furthermore, food processing such as pasteurization, sterilization, and storage could alter the structure of polyphenols [14], which show a diversity that is far beyond the well-known and abundant catechins. In fact, green tea contains, as other tea varieties, flavonol 3-*O*-glycosides [15,16], as well as other typical compounds such as theophylline, theanine, and theobromine [17]. Nevertheless, the content of the other polyphenolic components has long been disregarded, whereas great efforts have been made to prepare catechin-based supplements that are particularly enriched in epigallocatechin gallate (EGCG), which, among the other catechins, appears to be the most reactive substance. Indeed, although EGCG in a dose range level equal to 800–1600 mg seems to be well tolerated [18,19], adverse hepatotoxic effects, probably due to a GT catechins abuse, pose health concerns [20]. The European Food Safety Authority (EFSA), reviewing the conclusions of several studies aimed at evaluating the effects of EGCG on the liver, concluded that if the green tea beverage intake is generally safe, some precautions must be taken toward supplements, because many of them bring doses of catechins that can cause liver health problems [21,22]. In reality, the great variability of the preparation methods and the composition of the supplements means that the amount of catechins in these products can differ considerably between one product and another. In this context, Hu et al. [23], reviewing the scientific literature, highlighted that the large part of the toxicological studies lacks a detailed chemical characterization of the tested material. The lack of standardization (or the not full understanding) of the composition of the green tea preparations in marketed products adds further complexity to the risk assessment.

Herein, in order to compare the chemical composition of *Camellia sinensis* leaves of different origins in marketed products (obtained with different processing methods) and to mainly verify their metabolic profile in polyphenol compounds, beyond catechins and further antioxidant activity, six different green tea-based materials underwent ultrasound-assisted maceration (UAM) using ethanol as extracting solvent. In particular, Chinese gunpowder tea, three Japanese teas (Sencha, Bancha and Matcha), a commercial preparation in a filter bag, and one green tea catechin-enriched supplement were investigated. Gunpowder tea, a form of Chinese tea in which each leaf has been rolled into a small round pellet, and Japanese green teas, Sencha, Bancha, and Matcha teas were selected. Sencha is made from cultivated tea bushes of *Camellia sinensis* var. *sinensis* and it is commonly from the upper leaves and buds, whereas the lower more mature leaves are usually used for Bancha. Instead, Matcha is an unfermented tea that is steamed, dried, and ground into a milled green tea powder. The alcoholic extracts obtained were analyzed for their metabolomic profile by applying UHPLC HR MS/MS techniques. The extracts obtained were used for a preliminary bioactivity screening using DPPH (2,2′-diphenyl-1-picrylhydrazyl), ABTS (2,2’-azino-bis(3-ethylbenzothiazoline-6-sulfonic acid)), ORAC (oxygen radicals absorbance capacity), and thiobarbituric acid reactive substances (TBARS) assays.

## 2. Results and Discussion

### 2.1. Chemical Composition of Alcoholic Extracts from Commercialized Green Tea-Based Materials

**Figure 1:**
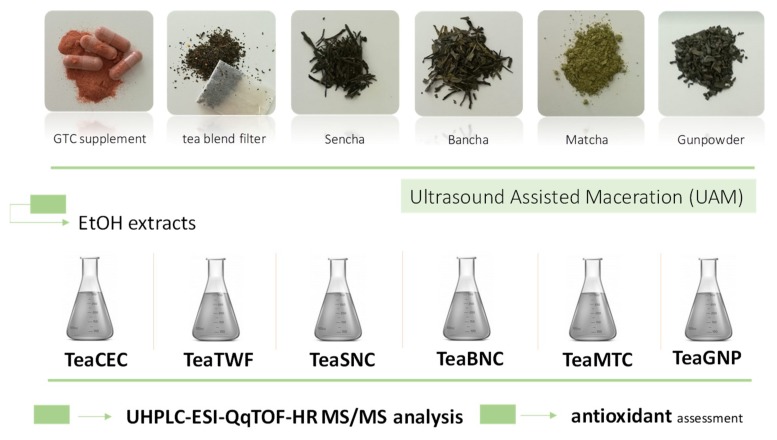
Extraction scheme applied on selected green tea matrices.

The catechin content of the selected green tea types were compared to that of a green tea catechin-based supplement, whereas the diversity in other phenolic, polyphenolic, and non-phenolic compounds was also evaluated. To this purpose, taking into account that ethanol is a good solvent for polyphenol extraction [24], also for higher molecular weight flavanols, alcoholic extracts were prepared by means of ultrasound-assisted maceration [25]. Previous investigation on black tea highlighted that the use of ultrasonic intensification led to an increase in polyphenols by approximately 15% in respect to conventional maceration [26]. Furthermore, it was observed that compared to the conventional shaking extraction methods, ultrasonic-assisted maceration on green tea leaves takes advantage of less extraction time and lower temperature [27]. The extraction scheme and the simple applied workflow are depicted in Figure 1.

The prepared extracts were investigated by means of ultra-high-performance liquid chromatography coupled with high-resolution mass spectrometry (UHPLC-HRMS). The total ion current chromatograms of extracts were mostly superimposable (Figure S1). As expected, flavanol compounds were the most representative, whereas flavonol substances, glycosylated and/or acylated, were also present in all the extracts but that obtained through ultrasound-assisted maceration on the green tea catechins-enriched supplement (TeaCEC). This latter almost completely lacked the saccharide and the great part of the flavonolic components. TeaCEC also did not contain theanine (**2**, Table 1), a non-protein amino acid contributing to the favorable taste [28,29], which was in a range of 0.34–0.57% in the other prepared extracts. The presence of this unique free amino acid was slightly more abundant in gunpowder green tea extract (TeaGNP, 0.54%) and Matcha tea extracts (TeaMTC, 0.57%). The molecule was tentatively identified on the basis of the deprotonated molecular ion at *m*/*z* 173.0932 and the fragment ion at *m*/*z* 155.0836, deriving from the neutral loss of a water molecule. The metabolites **1**, **4**, and **5** were saccharides (Figure S2, Table 1). In particular, the metabolite **1**, showing the ion [M − H]^−^ at *m*/*z* 311.0992, dissociated by supplying the ion at *m*/*z* 179.0560, due to a hexose unit, through the neutral loss of 132.0424 Da. This latter was according to a pentose residue. The presence of a pentose binding a hexose is also confirmed by the ion at *m*/*z* 161.0453. The metabolite **4** is putatively a trihexoside (e.g., raffinose). The ion [M − H]^−^ at *m*/*z* 503.1637 underwent the loss of two C_6_H_10_O_5_ moieties to provide the ions at *m*/*z* 341.1097 and 179.0558. The ion at *m*/*z* 341.1097 gave rise by the loss of two C_2_H_4_O_2_ units the fragment ions at *m*/*z* 281.0891 and 221.0667 (Figure S2). Compound **5** was putatively a dihexose or, as hypothesized based on TOF-MS^2^ spectrum, a mixture of dihexoses. In fact, it was observed that when sucrose co-eluted with turanose, fragment ions at *m*/*z* 143, 131, and 101 are detectable [30]. Both the disaccharides were previously reported as constituents of tea leaves and their abundance, together with that of maltose, cellobiose, and trehalose, appeared to be related to the tea plant growing condition as a response to cold stress [31].

Compounds **3** and **6** were tentatively identified as quinic acid and gallic acid, respectively, whereas the TOF-MS/MS spectrum of compound **7** was in accordance with theogallin, a depside of gallic acid and quinic one. Gallic acid and theogallin are the most abundant simple phenols in tea, and their relative content strongly depends on fermentation processes that favor the breakdown of the ester linkage, enhancing the gallic acid amount [32]. Indeed, gallic acid was found to be significantly released after enzymatic degradation during the fermentation process also from gallocatechin constituents [33]. Theogallin was suggested to slow down cognitive decline, improving brain health [34].

**Figure 2:**
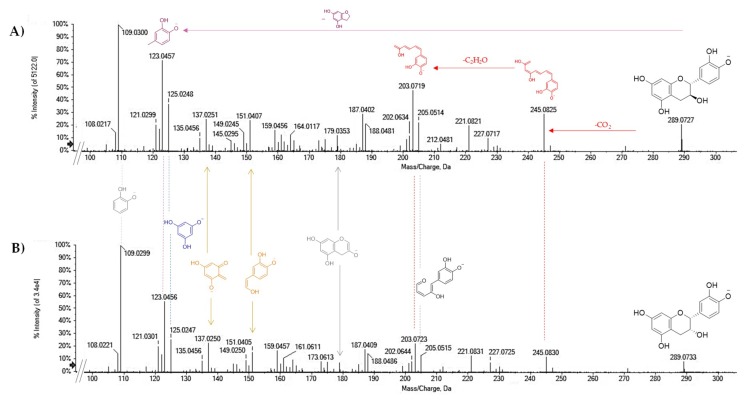
TOF-MS/MS spectra of [M − H]^−^ ion of compounds (**A**) **11** and (**B**) **14**. The structure of the main fragment ions is highlighted. The relative abundance of the ions [M − H-CO_2_]^−^ and [M − H-CO_2_-C_2_H_2_O]^−^, as well as of ions from benzofuran-forming fission (BFF, in purple), heterocyclic ring fission (HRF, in blue), and retro Diels–Alder (RDA, in light orange) reactions allowed the geometrical isomers to be distinguished.

The metabolites **8**–**11**, **14**, **15**, **17**, **23**, **26**, **34**, and **35** belong to the class of catechins (Table 2) and were identified as such on the basis of some of their characteristic peaks. Among the latter, the fragment ion at *m*/*z* 125.02, corresponding to the ring A of the flavanolic nucleus [35], is produced following the HRF (heterocyclic ring fission) reaction. The fragment ion, common to all catechins, represents the base peak of molecules **8** and **10**, which were identified on the basis of mass spectrometric data as gallocatechin isomers (Figure S3). Both the isomers were also characterized by the ions at *m*/*z* 167.0356(3) and 137.0251(48), which are produced by RDA (retro Diels–Alder) reaction. The two deprotonated ions at *m*/*z* 289.0722 for compounds **11** and **14**, eluted at different retention times, were in accordance with catechin and epicatechin diastereoisomers (Figure 2). The TOF-MS/MS spectra of both the molecules showed product ions at *m*/*z* 245.0825(30) by carbon dioxide loss and A-ring cleavage, and further ethenone loss to achieve ions at *m*/*z* 203.0719(23). The characteristic ions formed by reactions as HRF (at *m*/*z* 125.0248(7)) and RDA (at *m*/*z* 137.0251(0) and 151.0407(5)) were also observed. The base peak at *m*/*z* 109.0300(299) could be due to deprotonated catecholic moiety (B ring), whose loss as a neutral residue instead favored the formation of the fragment ion at *m*/*z* 179.0353(58). A benzofuran-forming fission (BFF) reaction provided the ion at *m*/*z* 123.0457(6).

Compound **9** was tentatively identified as (epi)gallocatechin dimer. The ion [M − H]^−^ at *m*/*z* 609.1265 was according to the C_30_H_26_O_14_ molecular formula. The neutral loss of 126.0344, due to the A-ring of a monomer, gave the ion at *m*/*z* 483.0950 through the HRF mechanism, whereas the ion at *m*/*z* 305.0665, corresponding to the monomeric unit, was from QM (quinone methide) fission with interflavanic bond cleavage. The RDA derived B-ring loss confirmed the presence of a pyrogallol moiety [36].

Compounds **15** and **17** were tentatively (epi)gallocatechin gallate isomers. The TOF-MS/MS spectra of both the compounds showed the loss of A-ring of the flavanolic unit (−126 Da), whereas the loss of the [gallic acid-H_2_O] moiety provided the fragment ion at *m*/*z* 305.0673(78). The presence of gallic acid was further confirmed by the fragment ion at *m*/*z* 169.0146(51), which was for compound **15** the base peak of the TOF-MS/MS spectrum (Figure S4A; [35,37]). A similar fragmentation pathway characterized compound **26**, which was tentatively identified as epicatechin gallate, whose TOF-MS/MS spectrum also showed the gallate ion as base peak (Figure S4B). Compound **35**, identified as epiafzelechin gallate, preferentially lost the dehydrated gallic acid residue, providing the ion at *m*/*z* 273.0777 as the base peak (Figure S5). The relative abundance of the fragment ion at *m*/*z* 183.0308(1) and the presence of the radical ion at *m*/*z* 168.0070 suggested for compounds **23** and **34** the occurrence of a methyl gallate bonded through ester bond to the alcoholic function in C-3 of epigallocatechin and epicatechin, respectively. All identified catechins are common to the extracts prepared with the exception of the metabolite **15**, which was detected only in the TeaGNP and TeaCEC samples. Epicatechin (**14**) is present in a slightly higher percentage in the Japanese Sencha green tea (TeaSNC), Bancha Japanese green tea (TeaBNC), and TeaMTC extracts, while metabolite **17** is more contained in TeaCEC (1.34-, 1.22-, 1.22-, 1.37- and 1.38-fold than Chinese green tea blend in filter (TeaTWF), TeaGNP, TeaSNC, TeaBNC, and TeaMTC, respectively). Epicatechin gallate is equally abundant in TeaCEC, where it was 1.6-fold more than that in TeaMTC.

Metabolites **12** and **13** were identified in chlorogenic acid (5-*O*-caffeoyl quinic acid, 5-CQA) and coumaroyl quinic acid [38,39]. If compound **12** is present only in traces in TeaCEC, the metabolite **13**, most contained in TeaGNP, was completely absent in the extract obtained from the supplement. Indeed, 5-CQA was identified as the main chlorogenic acid in tea, and its content was found to vary depending on the type of tea (white, green, black tea, and mate) and the processing technology [40].

The other identified metabolites belong to the class of glycosylated flavones and flavonols (Table 3). These substances, which resulted in trace amounts or complete absence in TeaCEC, mainly showed quercetin and kaempferol as aglycone moiety in mono-, di- and tri-saccharidic compounds. In particular, the metabolites **16** and **19** are the di-C-glycoside of the apigenin flavone (Figure S6). The TOF-MS/MS spectrum of the ion [M − H]^−^ at *m*/*z* 593.1555 for compound **16** underwent the loss of 90 and 120 Da according to sugar moiety cross-ring linkage [41], whereas the TOF-MS/MS ion at *m*/*z* 563.1442 for **19** gave ions at *m*/*z* 503.1225 [M − H-60]^−^, 473.1113 [M − H-90]^−^, and 443.1004 [M − H-120]^−^. These losses allowed us to hypothesize the presence of saccharide residues linked by the C-C bond [42]. The fragment ions at *m*/*z* 383 [aglycone + 113]^−^ and 353 [aglycone + 83]^−^ were in accordance with the presence of apigenin as aglycone. Different flavone C-glycosides were previously identified in green tea, and apigenin 6-C-arabinosyl-8-C-glucoside (isoschaftoside) and apigenin 6-C-glucosyl-8-C-arabinoside (schaftoside) were identified among this class of compounds [43]. The relative abundance in TOF-MS/MS spectrum of compound **19** of the ion at *m*/*z* 443 and the absence of 273 ion was in accordance with isoschaftoside occurrence.

**Figure 3:**
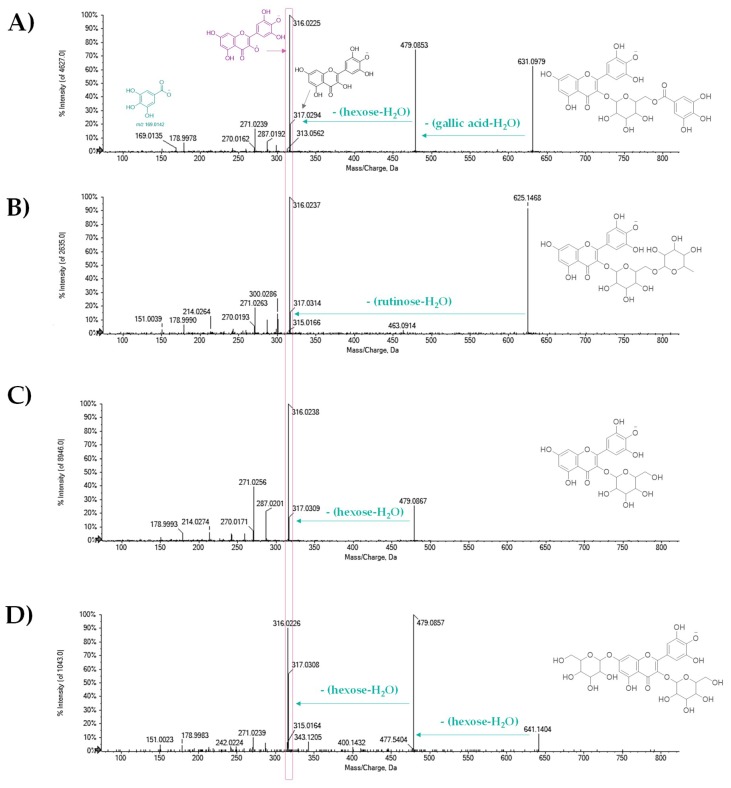
TOF-MS/MS spectra of [M − H]^−^ ion of myricetin glycosides (**A**) **18**, (**B**) **20**, (**C**) **21**, and (**D**) **22**. The structure of the deprotonated molecular ion is reported on the left, whereas the main neutral losses are evidenced by green arrays.

**Figure 4:**
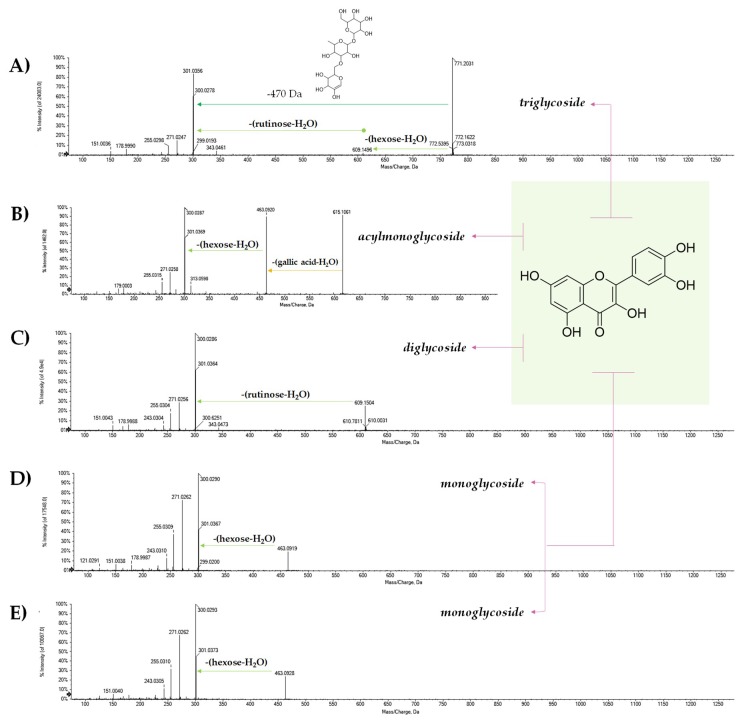
TOF-MS/MS spectra of [M − H]^−^ ion of quercetin glycosides (**A**) **24**, (**B**) **25**, (**C**) **27**, (**D**) **28**, and (**E**) **30**.

**Figure 5:**
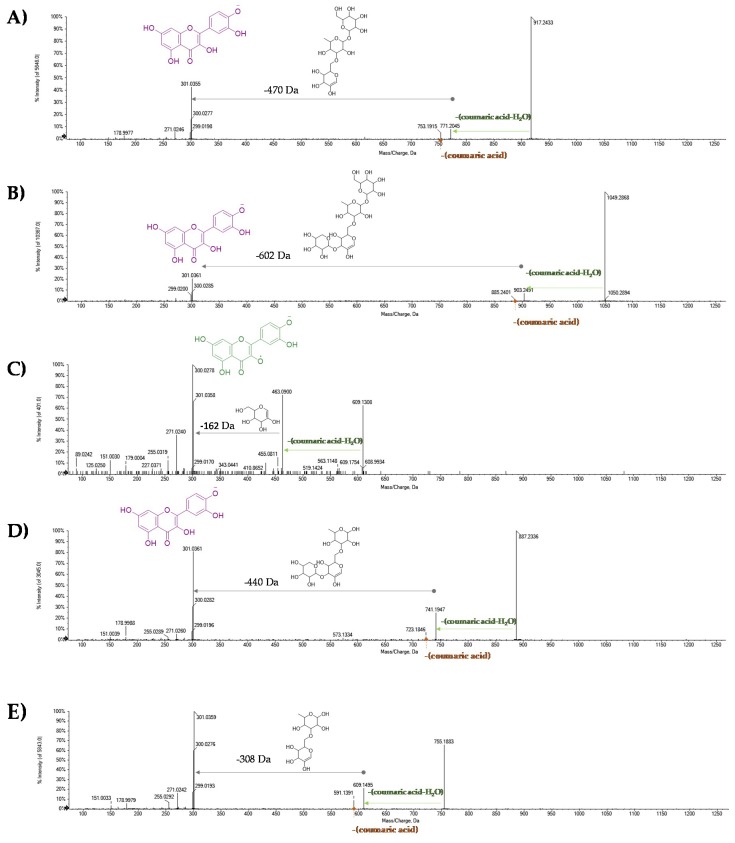
TOF-MS/MS spectra of [M − H]^−^ ion of some of tentatively identified quercetin coumaroyl glycosides (**A**) **36**, (**B**) **37**, (**C**) **38**, (**D**) **39**, and (**E**) **40**.

The metabolites **21**, **28**, **30**, **31**, and **33** have been identified as monoglycosyl derivatives of flavonols myricetin (**21**, [44]), quercetin (**28** and **30**), kaempferol (**31**), and methylkaempferol (**33**). The TOF-MS/MS spectrum of compound **21** is in Figure 3C, whereas the TOF-MS/MS spectra of the two quercetin hexosides are reported in Figure 4 (panels D and E). In all cases, the loss of the dehydrated hexose residue led to the formation of radical aglycone as the base peak, leaving the localization of the saccharide unit in C-3 of the aglycone to be hypothesized. The presence of hexose derivatives of the three flavonols—myricetin, quercetin, and kaempferol—has long been reported in tea leaves. Takino et al. [45] reported that hexose sugars could consist of glucose and its epimers galactose and mannose. Compound **18** with the [M − H]^−^ ion at *m*/*z* 631.0950 was tentatively identified as myricetin galloylhexoside (Figure 3A). To strengthen this hypothesis, the loss of 152 Da provided the fragment ion at *m*/*z* 479.0853, which gave rise to the aglycone anion at *m*/*z* 317.0294, which is attributable to myricetin, and its aglycone radical anion at *m*/*z* 316.0225. The abundance of this latter favored the hexose moiety to be localized at the C-3 carbon, as for compound **21** (Figure 3C). The acyl derivatives of flavonol monoglycoside were further compounds **25**, **38**, and **42**. Compound **25** showed neutral losses similar to that of compound **18**, according to quercetin galloylhexoside (Figure 4B). Compounds **38** and **42** were tentatively identified, based on their TOF-MS/MS spectra, in a quercetin coumaroylhexoside and kaempferol coumaroylhexoside, respectively. The [M − H]^−^ ion of compound **38** at *m*/*z* 609.1258 (Figure 5C) was in accordance with the molecular formula C_30_H_26_O_14_, whereas the deprotonated molecular ion of compound **42** suggested the molecular formula C_30_H_26_O_13_. The loss of dehydrated coumaric acid was ascertained through TOF-MS/MS experiments.

Compounds **27** and **32** were putatively identified as quercetin-3-*O*-rutinoside (Figure 4C) and kaempferol-7-O-rutinoside, respectively. Compounds **20** and **22** were tentatively identified as myricetin diglycosides, differing in the glyconic moiety, which appeared to be constituted by a rutinosyl residue in compound **20** (Figure 3B). Two hexose residues characterized compound **22**, which, based on the high abundance of the ion at *m*/*z* 479.0857, derived from the loss of the first hexosyl moiety, were each hypothesized to be located on C-3 or C-7 carbons (Figure 3D).

The remaining compounds present a greater complexity of the non-aglyconic portion. In particular, compound **24**, showing the deprotonated molecular ion at *m*/*z* 771.2004, in accordance with the molecular formula C_33_H_40_O_21_, provided, in the TOF-MS^2^ experiment, the [aglycone-H]^−^ ion and the most abundant radical aglycone, consisting with the loss of a triglycoside residue linked to the -OH enol group in C-3 of quercetin, whose identity was established on the basis of the detection of ions [M − H-CH_2_O]^−^ and [M − H-CH_2_O_2_]^−^ at *m*/*z* 271.0247 and 255.0298, respectively (Figure 4A). The TOF-MS/MS spectrum of compound **29** was in agreement with the presence of a kaempferol trisaccharide. Accordingly, previous studies suggested the occurrence of a kaempferol triglycoside in which the saccharide moiety consisted of (3”-*O*-galactosyl)rutinose [46]. The content of these molecules was more abundant in the TeaMTC sample. Recent studies have also found triglycosides of myricetin and have defined their disappearance as a consequence of the fermentation and oxidation processes [46]. Recently, the obtainment of a flavonol glycosides-rich extract named FLG was optimized from an aqueous green tea extract after tannase treatment [47]. Apigenin-C-mono- and diglycosides, as well as some different mono- and diglycosides of flavonols myricetin, quercetin, and kaempferol were detected.

**Figure 6:**
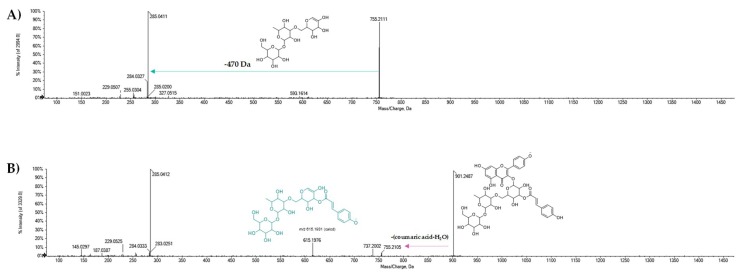
TOF-MS/MS spectra of [M − H]^−^ ion of some of kaempferol triglycosides (**A**) **29** and (**B**) **41**. In the B panel, the presence of a coumaroyltryglycosyl moiety was highlighted by detecting the ion at *m*/*z* 615.1976.

The metabolites **36**, **37**, **39**, and **40** were further identified as acylglycosides of the flavonol quercetin (Figure 5). In particular, the [M − H]^−^ ion of metabolite **36** at *m*/*z* 917.2361 dissociated by supplying the fragment ions at *m*/*z* 771.2075 and 753.1915, according to the neutral loss of a residue of coumaric acid-H_2_O and coumarate (Figure 5A). This compound was recognized as camelliquercitoside B. The [M − H]^−^ ion at *m*/*z* 1049.2789 for compound **37** was in line with camelliquercitoside A occurrence, whereas compound **39** could be camelliquercitoside C. In all the TOF-MS/MS spectra of these compounds, the [M − H]^−^ ion dissociated providing both the [M − H-coumaric acid-H_2_O]^−^ and [M − H-coumaric acid]^−^ ions, whose ratio appeared to vary depending on saccharide moiety. This finding was in line with a common localization of the hydroxycinnamoyl moiety on the sugar component directly linked to the aglycone. Instead, compound **40** was tentatively identified as camelliquercitoside D (Figure 5E). The [M − H]^−^ ion underwent a loss of coumaroyl moiety to achieve the ion at *m*/*z* 609.1495, which was likely rutin. Finally, compound **41** was putatively a coumaroyl derivative of compound **29** (Figure 6). In fact, the detected [M − H-coumaric acid-H_2_O]^−^ ion at *m*/*z* 755.2105 could lose 470 Da to provide the deprotonated kampferol aglycone. It was putatively identified as camellikaempferoside C, which is an acylated flavonol tetraglycoside that was recently isolated from Lu’an GuaPian green tea [48]. The identification of acylated flavonol di- and tryglycosides in different tea types is quite recent. In particular, flavonol mono- and diglycosides were reported as functional food ingredients that are able to regulate postprandial hyperglycemia through the inhibition of α-glucosidase/α-amylase [49].

Kaempferol coumaroyl glycosides, such as camellikaempferoside B, were reported as a constituent of Fuzhuan brick tea, which is a kind of uniquely microbial fermented tea in China [50]. This compound was found to possess the properties of both kaempferol and p-coumaric acid and significantly inhibit Aβ production by decreasing β-secretase activity [51]. Their biosynthesis is mediated by an acyltransferase, namely CsHCT, that can catalyze the transfer of an acyl group to the donor substrate [52]. Acyl flavonoid glycosides were identified as active ingredients with good inhibitory abilities on α-glucosidase and HMG-CoA reductase, and the hypoglycemic and hypolipidemic effects of Fuzhuan brick tea were attributable to their presence [53].

### 2.2. Estimation of Radical Scavenging, Antioxidant, and Antilipoperoxidant Activity

**Figure 7:**
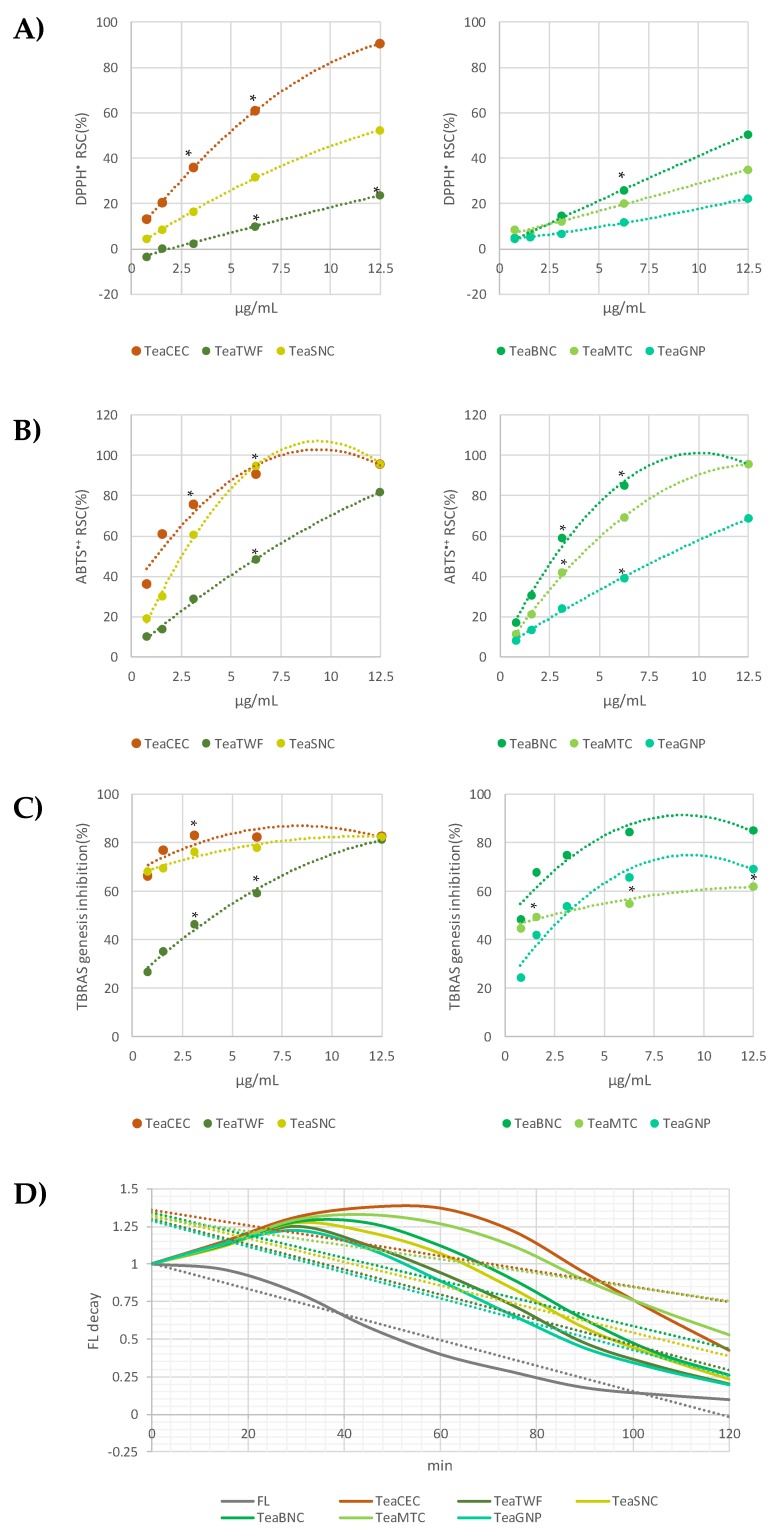
Radical Scavenging Capacity (RSC, %) of green tea extracts vs. 2,2′-diphenyl-1-picrylhydrazyl) (DPPH) radical (**A**) and 2,2’-azino-bis(3-ethylbenzothiazoline-6-sulfonic acid (ABTS) radical cation (**B**). Inhibition of thiobarbituric acid reactive substances (TBARS) species formation (%; **C**). Values are the mean ± SD of two independent experiments performed in triplicate. *p < 0.05 vs. blank. (**D**) Fluorescein fluorescence decay induced by the 2,2′-azobis-(2-amidinopropane)-dihydrochloride (AAPH) radical generator. The decay is strongly delayed by the co-presence of the extracts already at 0.78 µg/mL dose.

Alcoholic green tea extracts were also compared for their antiradical and antilipoperoxidant capabilities, although aware that, on the basis of the chemical composition, the TeaCEC extract, presenting the non-flavonoid component only in traces and completely lacking in the flavonol glycosides, could exert a more marked efficacy in the employed test tubes. In fact, tea catechins were broadly investigated for their antioxidant properties, and several studies defined the structural variants responsible for this activity [54]. Catechins reactivity in cell-free systems is attributable to the catechol/pyrogalloyl function on the B-ring, and their antiradical efficacy is greatly increased by esterification with a residue of gallic acid [55,56]. Indeed, large discrepancies are found in the literature among different green teas, suggesting that technological processes such as wilting, twisting, or drying, mostly affect the antioxidant properties of the product that reaches the consumer [57]. Thus, the co-presence in the obtained extracts of flavonolic glycosylated molecules and saccharides favored the comparative execution of four widely employed tests in the chemical antioxidant scenario. The anti-free radical tests DPPH^•^ and ABTS^+•^ were based on a mixed mechanism of action, given by the ability of a molecule to inactivate radical species with mechanisms of transfer of a hydrogen atom (HAT) or a single electron (SET). Data acquired, which clearly showed the dose-dependent radical scavenging action of the tested samples, are graphed in Figure 7 (panels A and B). The results from the most responsive ABTS assay evidenced that TeaCEC was able to almost completely reduce the probe species at a 3.125 µg/mL dose level; meanwhile, the TeaBNC and TeaSNC samples exerted a comparable reducing effect. Based on DPPH and ABTS ID_50_ values, it could be stated that the antiradical activity is negatively affected by the content of saccharide and quinic acid in the extracts.

The TBARS assay further demonstrated that green tea extracts effectively counteracted a UV-induced lipoxidation of hemp oil, which is highly rich in Polyunsaturated Fatty Acids (PUFA), and therefore highly unstable (Figure 7C). Data obtained were in line with the results by Lotito and Fraga [58], who, analyzing the effectiveness of pure catechins, found that they were effective antioxidants in human blood plasma, being able to delay lipoperoxidation and depletion of fat-soluble endogenous antioxidants such as β-carotene and α-tocopherol. Finally, an ORAC test also confirmed that all the extracts were able to prevent the formation of peroxyl radicals causing an increase in the fluorescein fluorescence. Curves obtained by the co-exposure of the radical generator, fluorescein, and extracts prepared at the lowest tested dose are in Figure 7D. TeaGNP and TeaTWF again appeared to be the less active extracts. Indeed, while TGP showed an antiradical trend closer to the other extracts, the TWF behavior was totally different. In spite of what was observed for the other two tests aimed at estimating the anti-free radical activity, TeaSNC was slightly less active than TeaBNC, and both are less effective than TeaMTC and TeaCEC.

The relative abundance of each class of compounds reasonably affects the antioxidant response. Catechin content in the partially purified TeaCEC extract reached the 89.9%, and it was 1.60-, 1.66-, 1.57-, 1.51-, 1.86-fold more abundant than in TeaTWF, TeaMTC, TeaSNC, TeaBNC, and TeaGNP, whereas its sugar amount was 14.1- and 13.0-fold lower than that in the less active TeaTWF and TeaGNP extracts. The higher relative content in catechins and chlorogenic acids of TeaBNC reasonably favored, in spite of its important sugar rate (11.0%), a good antiradical and antilipoperoxidant activity. It is noteworthy to be highlighted that TeaSNC, which showed a catechin content similar to that of TeaBNC, accounted for almost half of the sugars. The Relative Antioxidant Capacity Index (RACI), whose calculation represents the average of the standard scores obtained from the raw data for the various methods, comprehensively emphasized that, taking into account TeaCEC exception, TeaSNC was more active than the others (Table 4).

**Figure 8:**
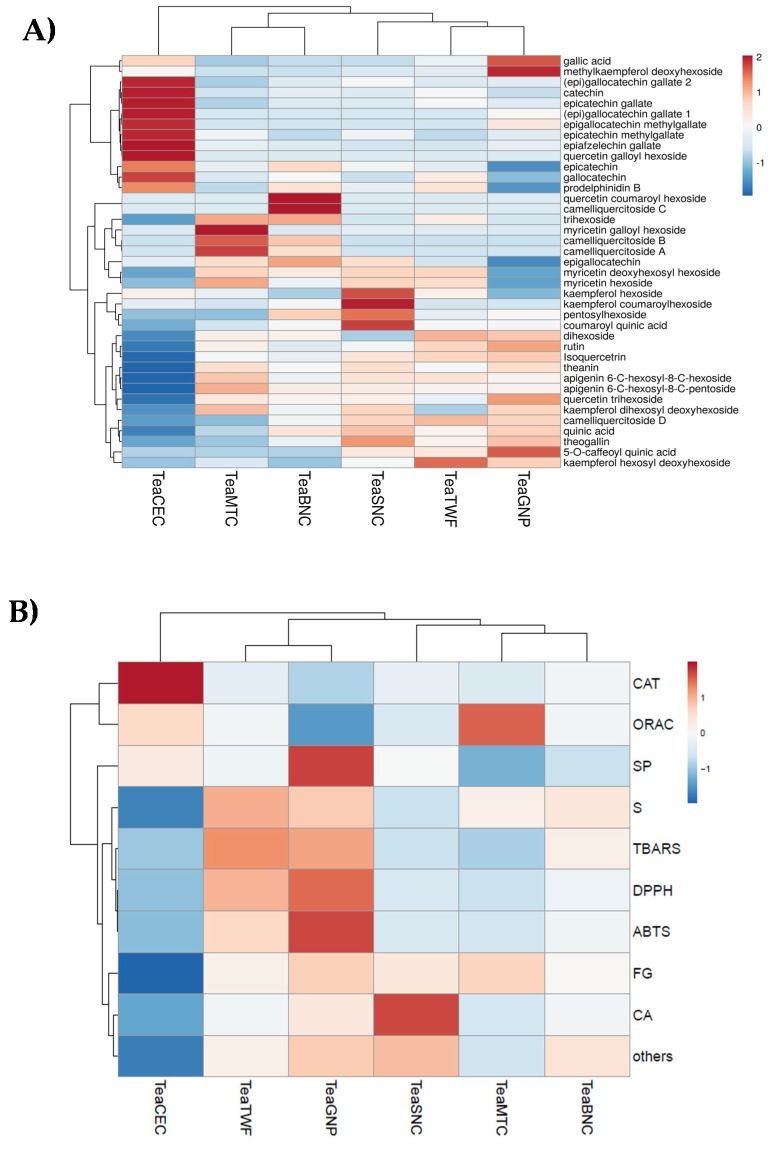
Heatmap (**A**) of compounds tentatively identified in alcoholic tea extracts; (**B**) relative content of each class of compounds (S = Sugars, SP = Simple Phenols; CA = Chlorogenic Acids; CAT = Catechins; FG = Flavonol Glycosides; “others” is for theanin and quinic acid). Annotations on top of the heatmap show clustering of the tea samples. In the ClustVis hierarchical clustering tool, both rows and columns are clustered using correlation distance and average linkage [59].

To get an overview, hierarchical clustering analysis was also applied using antiradical and antilipoperoxidant data, as well as the relative content of each class of compounds in investigated extracts (Figure 8). As mentioned above, differences in composition and therefore in bioactivity may depend on the different geographical location of the plantations and on the different methods of processing and refinement of the leaf. There is no information on conservation methods before purchase, but unpredictable exposure to heat or incorrect storage can lead to the destruction of some polyphenolic and non-polyphenolic compounds, or to epimerization reactions [13].

## 3. Materials and Methods

### 3.1. Green Tea Samples and Preparation of their Alcoholic Extracts

Commercial green tea leaves from different geographical areas and processing were the focus of the present investigation. All the matrices were bought from a supermarket or herbalist’s shop. In particular, Chinese green tea blend in filter (throughout the text referred to as TeaTWF), Chinese gunpowder green tea (TeaGNP), Japanese Sencha green tea (TeaSNC), Japanese Bancha green tea (TeaBNC) and Japanese Matcha green tea (TeaMTC) were analyzed together along with the green tea-based supplement (TeaCEC), which was reported to be titrated to 80% of total catechins, of which 50% were EGCG.

Tea samples (10 g each) underwent ultrasound accelerated maceration (Branson UltrasonicsTM Bransonic^TM^ M3800-E, Danbury, CT, USA), using ethanol as the extracting solvent in a ratio tea leaf:solvent equal to 1:5. In order to obtain the complete recovery of the tea leaf metabolic content, five sonication cycles were performed (30 min each) on all the tea samples, except for MTC, which was subjected to four sonication cycles, and CEC, whose catechins’ recovery was after one only cycle. At the end of each sonication cycle, samples were centrifuged at 3500× *g* for 10 min at 4 °C and then filtered using a paper filter. The extracts obtained were dried under vacuum using a rotary evaporator (Heidolph Hei-VAP Advantage, Schwabach, Germany).

### 3.2. UHPLC-ESI-QqTOF-MS and MS/MS Analyses

The alcoholic tea extracts, reconstituted in methanol LC-MS grade, at a 10 mg/mL dose level, were analyzed using a Shimadzu NEXERA UHPLC system (Shimadzu, Tokyo, Japan) equipped with a Luna^®^ Omega Polar C18 column (1.6 μm, 50 × 2.1 mm i.d, Phenomenex, Torrance, CA, USA). The separation was achieved using a binary solution A) H_2_O (0.1% HCOOH), B) CH_3_CN (0.1% HCOOH) using a gradient program, which started at 5% B, held for 1.5 min, and linearly ramping up to 25% B in 11 min. Then, the initial condition was restored and held for another 0.5 min. The total run time was 13 min, with a flow rate of 0.4 mL min^−1^. The injection volume was 2.0 μL.

MS analysis was performed using a hybrid Q-TOF MS instrument, the AB SCIEX Triple TOF^®^ 4600 (AB Sciex, Concord, ON, Canada), equipped with a DuoSprayTM ion source (consisting of both electrospray ionization (ESI) and atmospheric pressure chemical ionization (APCI) probes), which was operated in the negative ESI mode. The APCI probe was used for automated mass calibration using the Calibrant Delivery System (CDS, AB Sciex, Concord, ON, Canada). The CDS injects a calibration solution matching the polarity of ionization and calibrates the mass axis of the Triple TOF^®^ system in all scan functions used (MS and/or MS/MS). The Q-TOF HRMS method, which combines TOF-MS and MS/MS with information-dependent acquisition (IDA) for identifying non-targeted and unexpected compounds, consisted of a full scan TOF survey (dwell time 100 ms, 100–1500 Da) and eight IDA MS/MS scans (dwell time 100 ms, 80–1250 Da). The MS parameters were as follows: curtain gas (CUR) 35 psi, nebulizer gas (GS 1) 60 psi, heated gas (GS 2) 60 psi, ion spray voltage (ISVF) 4.5 kV, interface heater temperature (TEM) 600 °C, declustering potential (DP) −75 V. Collision Energy (CE) applied was −45 V with a collision energy spread (CES) of 15 V. The instrument was controlled by Analyst^®^ TF 1.7 software (AB Sciex, Concord, ON, Canada, 2016), while data processing was carried out using PeakView^®^ software version 2.2 (AB Sciex, Concord, ON, Canada, 2016).

### 3.3. Assessment of the Antioxidant Effectiveness

Investigated green tea extracts were tested at different dosage levels. Tests were carried out for three replicate measurements each. Recorded activities were compared to a blank treated equally to the samples. The assessment of antioxidant and radical scavenging abilities of investigated tea extracts was carried through four methods. Each extract was estimated at dose levels equal to (0.78, 1.56, 3.125, 6.25, and 12.5 μg/mL—final concentration levels), and its activity was compared to a blank arranged in parallel to the samples. ABTS [2,2′-azinobis-(3-ethylbenzothiazolin-6-sulfonic acid)] radical cation scavenging capacity and 2,2-diphenyl-1-picrylhydrazyl (DPPH) radical scavenging capability were determined as previously reported. TBARS and ORAC methods were also applied. Results are the mean ± SD values. ID_50_ values were calculated.

#### 3.3.1. Determination of 2,2′-Diphenyl-1-Picrylhydrazyl (DPPH) Radical Scavenging Capacity

The determination of DPPH^●^ scavenging capability was estimated as follows: increasing doses of green tea extracts were dissolved in a DPPH^●^ methanol solution (9.4 × 10^−5^ M) at room temperature. After 15 min of incubation, the absorption at 517 nm was measured by a Wallac Victor3 spectrophotometer in reference to a blank. The results are expressed in terms of the percentage reduction of the initial DPPH radical adsorption by green tea test samples. Trolox (4, 8, 16, 32 μM) was used as standard [60].

#### 3.3.2. Determination of ABTS [2,2′-Azinobis-(3-Ethylbenzothiazolin-6-Sulfonic Acid)] Radical Cation Scavenging Capacity

The radical cation ABTS was previously generated by the reaction between the diammonium salt of the 2,2’-azinobis-3-etilbenzotiazolin- 6-sulfonic acid (ABTS) (7.0 mM) and potassium persulfate (K_2_S_2_O_8_) (2.45 mM), leaving the reaction mixture in the dark for 12 h. Subsequently, the solution containing the radical cation ABTS was diluted in PBS (phosphate-buffered saline) in order to obtain an absorbance of 0.7 at 734 nm. Increasing doses of green tea were dissolved in the ABTS^●+^ solution. After incubation (6 min) the absorbance was measured at 734 nm, using a Wallac Victor3 spectrophotometer (PerkinElmer, Waltham, MA, USA) in reference to a blank. Trolox (4, 8, 16, 32 μM) was used as standard [61].

#### 3.3.3. Determination of Thiobarbituric Acid Reactive Substances (TBARS)

Thiobarbituric acid (TBA) reagent was prepared as follows: for reagent A, TBA (375.0 mg) and tannic acid (30.0 mg) were dissolved in hot water (30.0 mL); for reagent B, trichloroacetic acid (15 g) was dissolved in an aqueous hydrogen chloride solution (0.30 M, 70.0 mL). Then, reagent A was mixed with reagent B. Next, hemp oil (6.0 μL) was emulsified with Tween-40 (15.6 mg) initially dissolved in Tris-HCl buffer (0.2 M, 2.0 mL, pH 7.4). Afterwards, 25 μL of each sample (0.78, 3.125, 6.25, 12.5, 25.0, 50.0 μg/mL; final concentration) and Trolox (2.0, 4.0, 8.0, 16.0 μM; final concentration) were added to 500 μL of reaction mixture. Samples were irradiated with UV light for 60 min at 254 nm. After addition of the TBA reagent (1.0 mL), all test tubes were placed in a boiling water bath for 15 min. Then, 300.0 μL of *n*-butanol were added and centrifuged for 3 min at 1500 g. The absorbance of supernatant was measured at 532 nm. The inhibition of lipid peroxidation was recorded as percentage versus a blank containing no test samples. Trolox (4, 8, 16, 32 μM) was used as standard [62].

#### 3.3.4. Determination of Oxygen Radical Absorbance Capacity (ORAC)

Green tea extracts (20 μL; 0.78, 1.56, and 3.125 μg/mL, final concentrations) and fluorescein (120.0 μL, 70 nM, final concentration) solutions were preincubated for 15 min at 37 °C in 75.0 mM phosphate buffer (pH 7.4). Then, 2,2′-azobis-(2-amidinopropane)-dihydrochloride (AAPH) solution (60.0 μL, 12.0 mM, final concentration) was rapidly added. In parallel with the samples, a blank (FL + AAPH) and solutions of the standard antioxidant Trolox (1–8 μM, final concentrations) were properly prepared in PBS. The fluorescence (λ_ex_ = 485 nm, λ_em_ = 525 nm) was recorded every 15 min for 120 min using a Wallac Victor3 (PerkinElmer, Waltham, MA, USA). Antioxidant curves (fluorescence versus time) were first normalized to the curve of the blank by multiplying original data by the factor fluorescence blank, t = 0/ fluorescence sample, t = 0. From the normalized curves, the area under the fluorescence decay curve (AUC) was calculated as follows: AUC = ∑ fi/f0, where f0 was the initial fluorescence reading at 0 min and fi was the fluorescence reading at time *i*. Linear regression equations between net AUC (AUC_antioxidant_ − AUC_blank_) and antioxidant concentration were calculated for all the samples [60].

### 3.4. Statistical Analysis

Antiradical and antilipoperoxidant assays were performed three times with replicate samples, except where otherwise indicated. Data are expressed as mean ± SD (standard deviation). The means were compared using analysis of variance (ANOVA) plus Bonferroni’s t-test. A *p*-value of < 0.05 was considered to indicate a statistically significant result. The Relative Antioxidant Capacity Index (RACI) was calculated according to the method of Sun and Tanumihardjo [63]. The standard score was calculated as follows: (x − μ)/σ where x is the raw data, μ is the mean, and σ is the standard deviation. Standard scores have a mean of 0 and standard deviation equal to 1. ClustVis, freely available at http://biit.cs.ut.ee/clustvis/, was used for data pre-processing and heatmap plotting.

## 4. Conclusions

Green tea, highly consumed worldwide, and with broadly proven properties, beyond catechins, is a reservoir of flavonol glycosides and their acylated derivatives. The content of these substances, together with that of chlorogenic acids, simple phenols, and saccharides is highly variable in marketed products, based on tea type and quality leaf. UAM extraction, together with HR MS/MS analyses, proved to maximize their disclosure, also when an extracting solvent that is not properly suitable for this class of compounds was used. The relative abundance of each class of compounds reasonably correlated to the antioxidant response. The antiradical activity was found to be negatively affected by saccharides and quinic acid amount in the extracts, in spite of the presence of catechins and/or other polyphenols. This finding was clearly appreciated when alcoholic extracts from TeaSNC and TeaBNC were compared. In fact, the first one proved to be more active than the second, although showing a similar catechin content but accounting for almost half of the sugars. The preventive role of flavonoid compounds makes green tea products, if properly standardized, a favorable tool to counteract oxidative stress conditions onset, in association with a healthy and correct lifestyle. Furthermore, the data acquired addresses the research for new extraction strategies that better lead to a full understanding of acylated flavonol glycosides content in other tea types. Moreover, the good solubility in water of flavonol glycosides and their acylated derivatives lays the foundation for identifying and quantifying them when herbal tea preparation is through call brewing.

## Figures and Tables

**Table 1:** TOF-MS and TOF-MS/MS of tentatively identified non-flavonoid compounds in investigated green tea extracts. Compounds are numbered based on their RT in the whole total ion current chromatogram. RT = Retention Time; RDB = Ring Double Bond equivalent value.

Peak	RT (min)	Tentative Assignment	Formula	[M − H]^−^ calc. (m/z)	[M − H]^−^ Found (m/z)	Error (ppm)	RDB	Fragment Ions MS/MS (m/z)
**1**	0.304	pentosylhexoside	C_11_H_20_O_10_	311.0984	311.0992	2.7	2	311.1004; 179.0560; 161.0453; 87.0086
**2**	0.310	theanin	C_7_H_14_N_2_O_3_	173.0932	173.0932	0.2	2	155.0836; 93.0350; 84.0459; 82.0313
**3**	0.316	quinic acid	C_7_H_12_O_6_	191.0561	191.0564	1.5	2	191.0564; 173.0451; 171.0292; 127.0399; 111.0451; 93.0347; 87.0087; 85.0298; 83.0500; 81.0344
**4**	0.319	trihexoside	C_18_H_32_O_16_	503.1618	503.1637 [M + Cl]^−^	3.7	3	503.1654; 341.1097; 323.0983; 281.0891; 251.0789; 221.0667; 179.0558; 161.0451; 119.0349; 113.0246; 89.0242
**5**	0.324	dihexoside	C_12_H_22_O_11_	341.1089	341.1102	3.7	2	341.1095; 179.0556; 161.0452; 149.0446; 143.0345; 131.0352; 119.0348; 113.0244; 101.0245; 95.0140; 89.0247
**6**	0.873	gallic acid	C_7_H_6_O_5_	169.0142	169.0148	3.3	5	125.0242
**7**	1.049	theogallin	C_14_H_16_O_10_	343.0671	343.0671	0.1	7	191.0561; 127.0397; 85.0290
**12**	2.515	5-*O*-caffeoyl quinic acid	C_16_H_18_O_9_	353.0877	353.0878	−0.3	8.0	191.0558; 179.0345; 173.0459; 135.0448; 85.0291
**13**	2.734	coumaroyl quinic acid	C_16_H_18_O_8_	337.0931	337.0929	0.6	8.0	191.0561; 173.0452; 163.0401; 119.0497; 93.0344

**Table 2:** TOF-MS and TOF-MS/MS of tentatively identified flavanols in investigated green tea extracts. Compounds are numbered based on their RT in the whole total ion current chromatogram. RT = Retention Time; RDB = Ring Double Bond equivalent value.

Peak	RT (min)	Tentative Assignment	Formula	[M − H]^−^ calc. (m/z)	[M − H]^−^ Found (m/z)	Error (ppm)	RDB	Fragment Ions MS/MS (m/z)
**8**	1.400	gallocatechin	C_15_H_14_O_7_	305.0667	305.0671	1.4	9	305.0678; 221.0463; 219.0672; 177.0561; 167.0356; 139.0407; 137.0251; 125.0253; 109.0300
**9**	1.771	prodelphinidin B	C_30_H_26_O_14_	609.1250	609.1265	2.5	18	609.1294; 483.0950; 441.0835; 423.0733; 355.0804; 305.0665; 255.0305; 221.0454; 179.0340; 177.0180; 125.0236
**10**	2.094	epigallocatechin	C_15_H_14_O_7_	305.0667	305.0671	1.4	9	305.0671; 219.0667; 167.0353; 139.0403; 137.0248; 125.0248; 109.0299
**11**	2.320	catechin	C_15_H_14_O_6_	289.0718	289.0722	1.5	9	289.0727; 245.0825; 221.0821; 205.0514; 203.0719; 187.0402; 179.0353; 151.0407; 137.0251; 125.0248; 123.0457; 109.0300
**14**	2.904	epicatechin	C_15_H_14_O_6_	289.0718	289.0722	1.5	9	289.0733; 245.0830; 221.0831; 205.0515; 203.0723; 187.0409; 179.0358; 151.0405; 137.0250; 125.0247; 123.0456; 109.0299
**15**	3.074	(epi)gallocatechin gallate 1	C_22_H_18_O_11_	457.0776	457.0786	2.1	14	331.0463; 305.0673; 219.0668; 193.0148; 169.0146; 161.0245; 137.0245; 125.0246
**17**	3.357	(epi)gallocatechin gallate 2	C_22_H_18_O_11_	457.0776	457.0788	2.5	14	331.0476; 305.0678; 219.0668; 193.0148; 169.0151; 161.0249; 137.0246; 125.0250
**23**	3.606	epigallocatechin methylgallate	C_23_H_20_O_11_	471.0943	471.0933	2.2	14	305.0678; 219.0667; 183.0308; 179.0355; 168.0070; 161.0251; 125.0249
**26**	3.691	epicatechin gallate	C_22_H_18_O_10_	441.0827	441.0836	2.0	14	441.0841; 331.0461; 303.0508; 289.0715; 245.0811; 203.0707; 193.0137; 169.0138; 151.0393; 137.0236; 125.0241; 124.0162; 109.0291
**34**	4.008	epicatechin methylgallate	C_23_H_20_O_10_	455.0995	455.0984	2.5	14	289.0724; 245.0819; 183.0301; 168.0070; 125.0242
**35**	4.018	epiafzelechin gallate	C_22_H_18_O_9_	425.0881	425.0883	0.7	14	425.0910; 287.0574; 273.0777; 255.0666; 243.0690; 211.0760; 169.0144; 125.0246; 97.0297

**Table 3:** TOF-MS and TOF-MS/MS of tentatively identified flavonol glycosides in investigated green tea extracts. Compounds are numbered based on their RT in the whole total ion current chromatogram. RT = Retention Time; RDB = Ring Double Bond equivalent value.

Peak	t_R_ (min)	Tentative Assignment	Formula	[M − H] ^−^ calc. (m/z)	[M − H]^−^ Found (m/z)	Error (ppm)	RDB	Fragment Ions MS/MS (m/z)
**16**	3.191	apigenin 6-C-hexosyl-8-C-hexoside	C_27_H_30_O_15_	593.1512	593.1520	1.4	13	593.1555; 575.1398; 545.1276; 503.1207; 473.1109; 395.0780; 383.0779; 353.0672; 325.0715; 297.0771; 221.0452; 191.0357
**18**	3.377	myricetin galloyl hexoside	C_28_H_24_O_17_	631.0941	631.0950	1.5	17	631.0979; 479.0853; 317.0294; 316.0225; 287.0192; 271.0239; 178.9978; 169.0135
**19**	3.480	apigenin 6-C-hexosyl-8-C-pentoside	C_26_H_28_O_14_	563.1406	563.1418	2.2	13	563.1442; 545.1324; 503.1225; 473.1113; 443.1004; 413.0889; 383.0782; 353.0672; 325.0722; 297.0771; 296.0685
**20**	3.497	myricetin deoxyhexosyl hexoside	C_27_H_30_O_17_	625.1410	625.1422	1.9	13	625.1468; 463.0914; 317.0314; 316.0237; 300.0286; 271.0263; 214.0264; 178.9990; 151.0039
**21**	3.503	myricetin hexoside	C_21_H_20_O_13_	479.0831	479.0847	3.3	12	479.0867; 317.0309; 316.0238; 287.0201; 271.0256; 214.0274; 178.9993
**22**	3.567	myricetin dihexoside	C_27_H_30_O_18_	641.1373	641.1359	2.1	13	641.1404; 479.0857; 317.0308; 316.0226; 271.0239; 178.9983; 151.0023
**24**	3.633	quercetin trihexoside	C_33_H_40_O_21_	771.1989	771.2004	1.9	14	771.2031; 609.1496; 343.0461; 301.0356; 300.0278; 271.0247; 255.0298; 178.9990; 151.0036
**25**	3.681	quercetin galloyl hexoside	C_28_H_24_O_16_	615.0992	615.0998	1.0	17	615.1061; 463.0920; 313.0598; 301.0369; 300.0287; 271.0258; 255.0315; 179.0003
**27**	3.703	rutin	C_27_H_30_O_16_	609.1461	609.1469	1.3	13	609.1504; 343.0473; 301.0364; 300.0286; 271.0256; 255.0304; 178.9988; 169.0146; 151.0043
**28**	3.819	Isoquercetrin	C_21_H_20_O_12_	463.0888	463.0887	*−*0.2	12	463.0919; 301.0367; 300.0290; 299.0200; 271.0262; 255.0309; 243.0310; 178.9987; 151.0038; 121.0291
**29**	3.825	kaempferol dihexosyl deoxyhexoside	C_33_H_40_O_20_	755.2040	755.2046	0.8	14	755.2111; 285.0411; 284.0327; 255.0304; 229.0507; 151.0023
**30**	3.842	quercetin hexoside	C_21_H_20_O_12_	463.0882	463.0887	1.1	12	463.0928; 301.0373; 300.0293; 271.0262; 255.0310; 243.0305; 151.0040
**31**	3.939	kaempferol hexoside	C_21_H_20_O_11_	447.0933	447.0941	2.0	12	285.0405; 284.0331; 255.0305; 227.0351; 211.0395; 183.0449
**32**	3.959	kaempferol hexosyl deoxyhexoside	C_27_H_30_O_15_	593.1531	593.1512	3.2	13	593.1553; 285.0407; 284.0329; 255.0301; 227.0349
**33**	4.007	methylkaempferol deoxyhexoside	C_21_H_20_O_11_	447.0933	447.0942	2.0	12	300.0291; 285.0409; 284.0333; 255.0303; 227.0353
**36**	4.386	camelliquercitoside B	C_42_H_46_O_23_	917.2357	917.2361	0.4	20	917.2433; 771.2075; 753.1915; 301.0355; 300.0277; 299.0198; 271.0246; 178.9977
**37**	4.419	camelliquercitoside A	C_47_H_54_O_27_	1049.2780	1049.2789	0.9	21	1049.2868; 903.2491; 885.2401; 301.0361; 300.0285; 299.0200
**38**	4.420	quercetin coumaroyl hexoside	C_30_H_26_O_14_	609.1250	609.1258	1.3	18	609.1308; 463.0900; 301.0358; 300.0278; 271.0240; 255.0319; 227.0371; 179.0004; 151.0030
**39**	4.447	camelliquercitoside C	C_41_H_44_O_22_	887.2251	887.2263	1.3	20	887.2336; 741.1947; 723.1846; 573.1334; 301.0361; 300.0282; 299.0196; 271.0260; 255.0289; 178.9988; 151.0039
**40**	4.550	camelliquercitoside D	C_36_H_36_O_18_	755.1829	755.1838	1.2	19	755.1883; 609.1495; 591.1391; 301.0359; 300.0276; 299.0193; 271.0242; 255.0292; 178.9979; 151.0033
**41**	4.553	camellikaempferoside C	C_42_H_46_O_22_	901.2408	901.2419	1.2	20	901.2487; 755.2105; 737.2002; 615.1976; 285.0412; 284.0333; 283.0251; 229.0525; 187.0387; 145.0297
**42**	4.654	kaempferol coumaroylhexoside	C_30_H_26_O_13_	593.1301	593.1325	4.1	18	593.1322; 447.0948; 285.0394; 284.0322; 255.0289; 227.0334; 145.0279

**Table 4:** Antioxidant activity of green tea extracts expressed as ID_50_ (μg/mL) and TEAC (Trolox^®^ Equivalent Antioxidant Capacity values, ID_50 Trolox_^®^ /ID_50 sample_) vs. DPPH and ABTS^+^ radicals. ID_50_ and Trolox^®^ Equivalent Antioxidant Capacity (TEAC) values from TBARS inhibition assay, as well as oxygen radical absorbance capacity (ORAC) values (Trolox^®^ Eq μM) are also reported. Relative Antioxidant Capacity Index (RACI) values, which were calculated taking into account the average of the standard scores obtained from the ID_50_ raw data for the various methods and ORAC values, are also given in the last column. TeaCEC: green tea catechins-enriched supplement, TeaTWF: Chinese green tea blend in filter, TeaSNC: Japanese Sencha green tea, TeaBNC: Japanese Bancha green tea, TeaMTC: Matcha green tea, TeaGNP: gunpowder green tea.

	ID_50_ DPPH (μg/mL) TEAC	ID_50_ ABTS (μg/mL) TEAC	ID_50_ TBARS (μg/mL) TEAC	ORAC (Trolox^®^ Eq μM)	RACI
**TeaCEC**	3.83 ± 0.25 3.91	1.13 ± 0.12 4.42	0.17 ± 0.01 38.2	3.71 ± 0.16	9.96
**TeaTWF**	66.52 ± 0.25 0.22	5.28 ± 0.72 0.95	3.14 ± 0.61 2.04	2.72 ± 0.16	−0.16
**TeaSNC**	15.37 ± 0.51 0.97	2.26 ± 0.32 2.20	0.28 ± 0.01 22.8	3.57 ± 0.63	5.62
**TeaBNC**	19.71 ± 0.38 0.76	2.44 ± 0.44 2.04	0.55 ± 0.04 11.5	2.53 ± 0.79	2.36
**TeaMTC**	31.15 ± 0.25 0.48	3.35 ± 0.53 1.49	1.79 ± 0.12 3.57	2.72 ± 0.32	0.32
**TeaGNP**	83.11 ± 0.15 0.18	7.80 ± 1.39 0.64	2.90 ± 0.56 2.20	2.02 ± 0.21	−0.44

## Supplementary Materials

The following are available online. Figure S1: Total Ion Chromatograms (TICs) of alcoholic extracts ● TeaTWF; ● TeaGNP; ● TeaSNC; ● TeaBNC; ● TeaMTC; ● TeaCEC. Figure S2: TOF-MS/MS spectra of deprotonated compound **1** (**A**), and [M + Cl]^−^ adducts of compounds **4** (**B**) and **5** (**C**). In the D panel, the formation of the ion at *m*/*z* 221 is proposed based on raffinose trihexose; *m*/*z* values, below each structure, are the calculated ones. Figure S3: TOF-MS/MS spectra of [M − H]^−^ ion of compound (**A**) **8**, and (**C**) **10**. In panel **B**, the fragmentation pattern that leads to the HRF-mediated formation of the fragment ion at *m*/*z* 125.0244 (calcd) is exemplified (in blue), whereas the RDA reaction gives the fragment ions at *m*/*z* 137.0244 e 167.0350 (calcd). This latter fragment ion is diagnostic for gallocatechins and all the derivatives showing a pyrogallol B-ring. Figure S4: TOF-MS/MS spectra of [M − H]^−^ ion of compound (**A**) **15**, and (**B**) **26**. The structure of the main fragment ions is highlighted; *m*/*z* values, below each structure are the calculated ones. Figure S5: TOF-MS/MS spectrum of [M − H]^−^ ion of compound **35**. The neutral loss of dehydrated gallic acid residue is highlighted; *m*/*z* values, below each structure, are the calculated ones. Figure S6: TOF-MS/MS spectra of [M − H]^−^ ion of compound (**A**) **16** and (**B**) **19**. The structures assigned to the compounds are reported in the grey panel.
