# Supplementary material for: Coumaroyl Flavonol Glycosides and More in Marketed Green Teas: An Intrinsic Value beyond Much-Lauded Catechins
Source: Molecules. 2020 Apr 11;25(8):1765. doi: 10.3390/molecules25081765 (PMC7221963; doi:10.3390/molecules25081765)

*Supplementary Material*

# Coumaroyl Flavonol Glycosides and more in marketed green teas: an intrinsic value beyond much-lauded catechins

**Lorenzo Candela, Marialuisa Formato, Giuseppina Crescente, Simona Piccolella, and Severina Pacifico***

**Figure S1.** Total Ion Chromatograms (TICs) of alcoholic extracts ● TeaTWF; ● TeaGNP; ● TeaSNC; ● TeaBNC; ● TeaMTC; ● TeaCEC

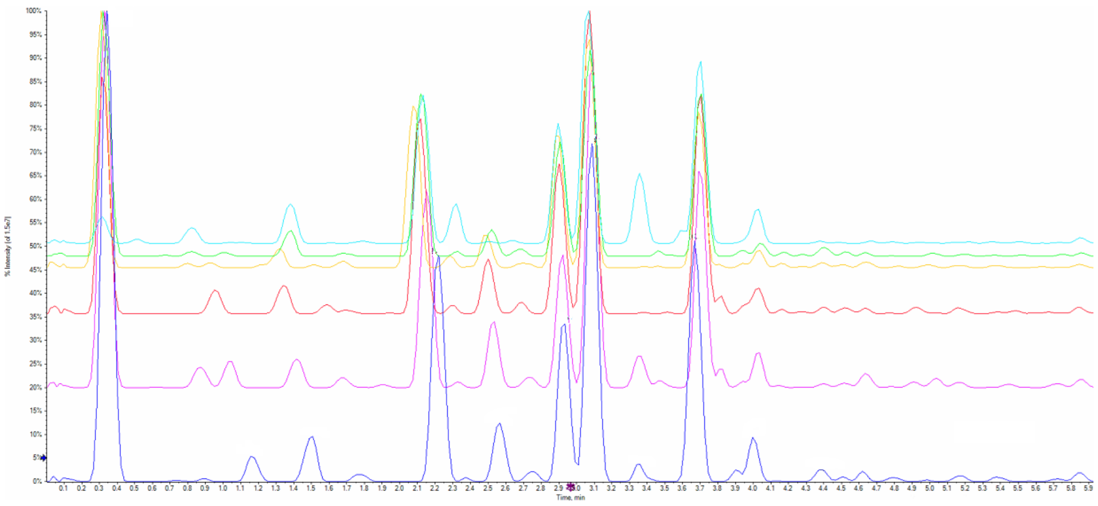

**Figure S2.** TOF-MS/MS spectra of deprotonated compound **1 (A)**, and [M+Cl]- adducts of compounds **4 (B)** and **5 (C)**. In D panel, the formation of the ion at *m*/*z* 221 is proposed based on raffinose trihexose; *m*/*z* values, below each structure, are the calculated ones

**A)**

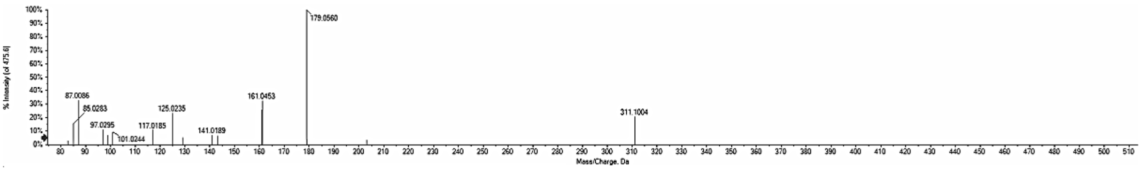


**B)**

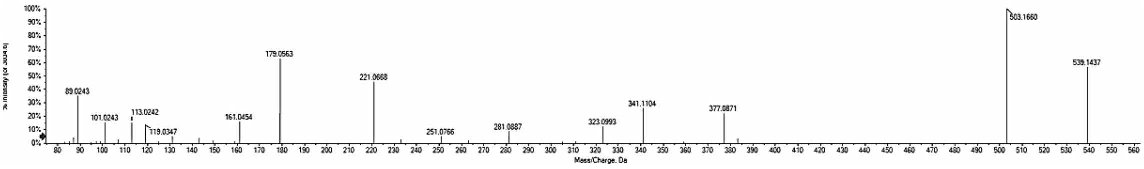


**C)**

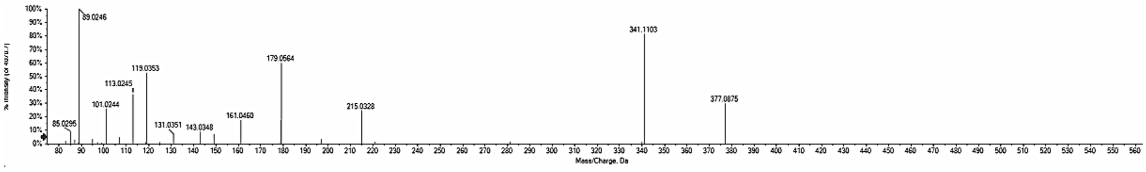


**D)**

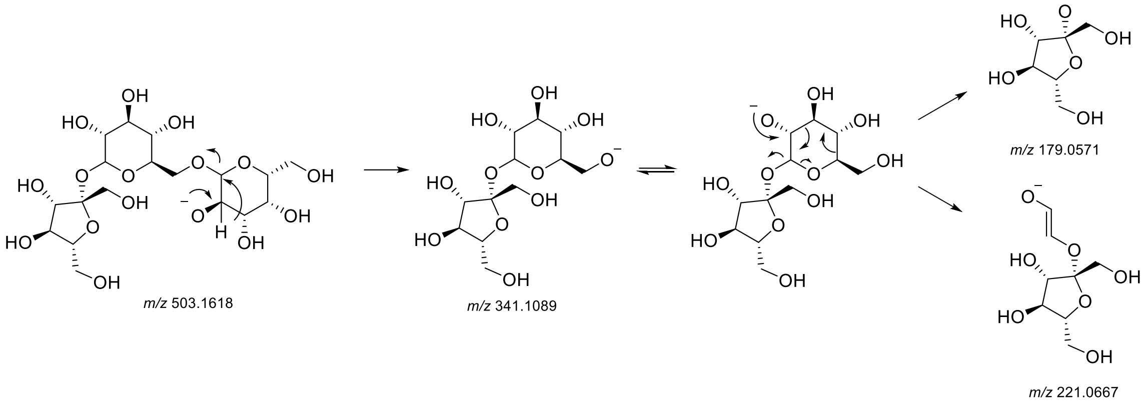

**Figure S3.** TOF-MS/MS spectra of [M-H]$^{-}$ ion of compound **A) 8**, and **C) 10**. In panel B the fragmentation pattern that leads to the HRF-mediated formation of the fragment ion at *m/z* 125.0244 (calcd) is exemplified (in blue), whereas RDA reaction gives the fragment ions at *m/z* 137.0244 e 167.0350 (calcd). This latter fragment ion is diagnostic for gallocatechins and all the derivatives showing pyrogallol B-ring.

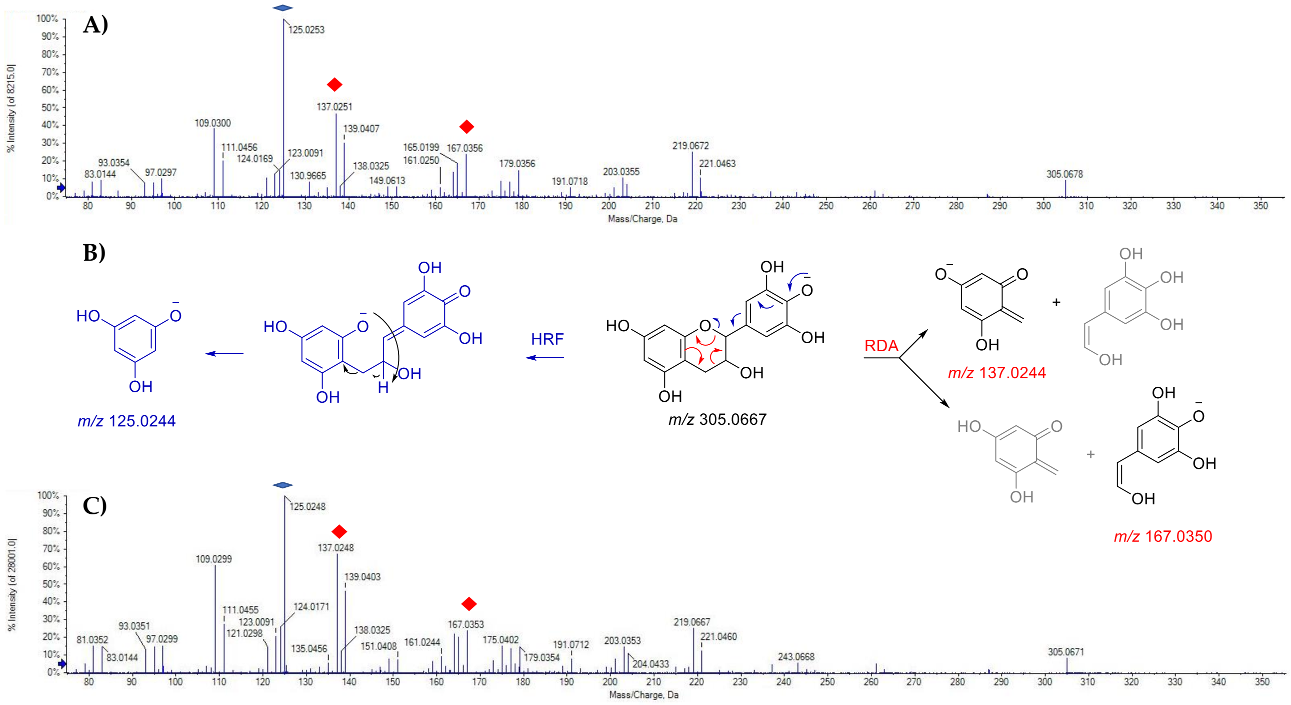

**Figure S4.** TOF-MS/MS spectra of [M-H]$^-$ ion of compound **A) 15**, and **B) 26**. The structure of the main fragment ions is highlighted; *m*/*z* values, below each structure, are the calculated ones.

A)

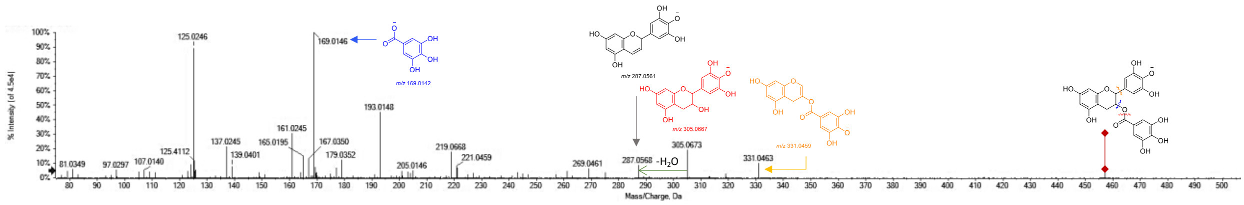


B)

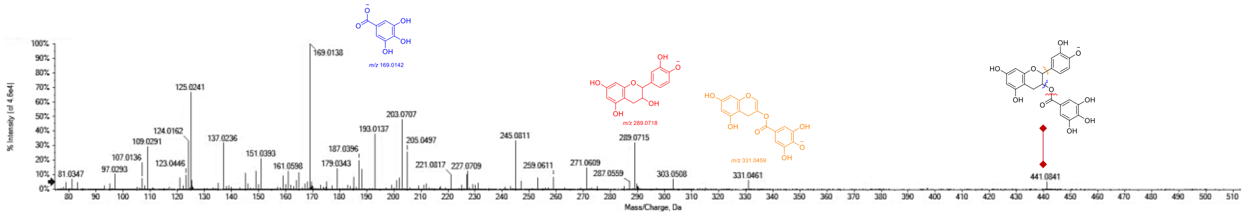

**Figure S5.** TOF-MS/MS spectrum of [M-H]⁻ ion of compound **35**. The neutral loss of dehydrated gallic acid residue is highlighted; *m*/*z* values, below each structure, are the calculated ones.

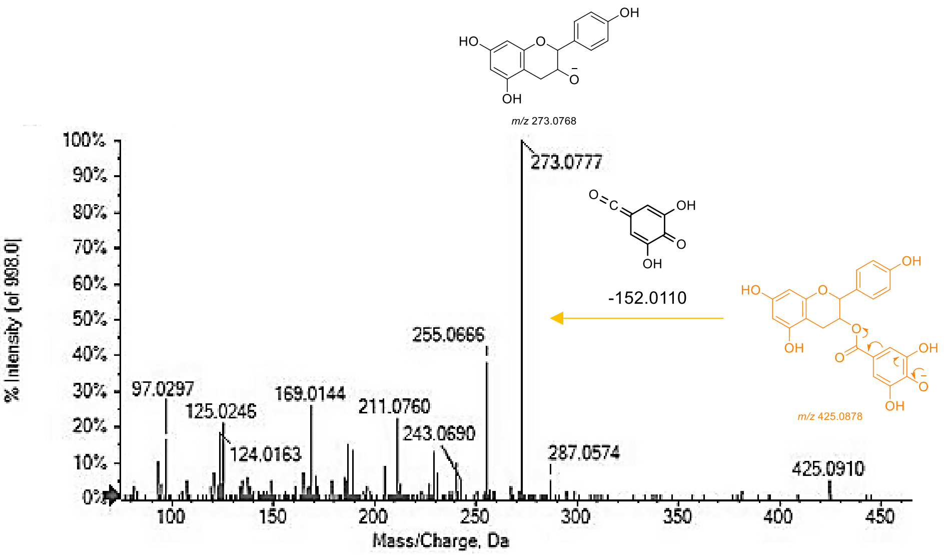

**Figure S6.** TOF-MS/MS spectra of [M-H]$^-$ ion of compound **A) 16**, and **B) 19**. The structures assigned to the compounds are reported in the grey panel.

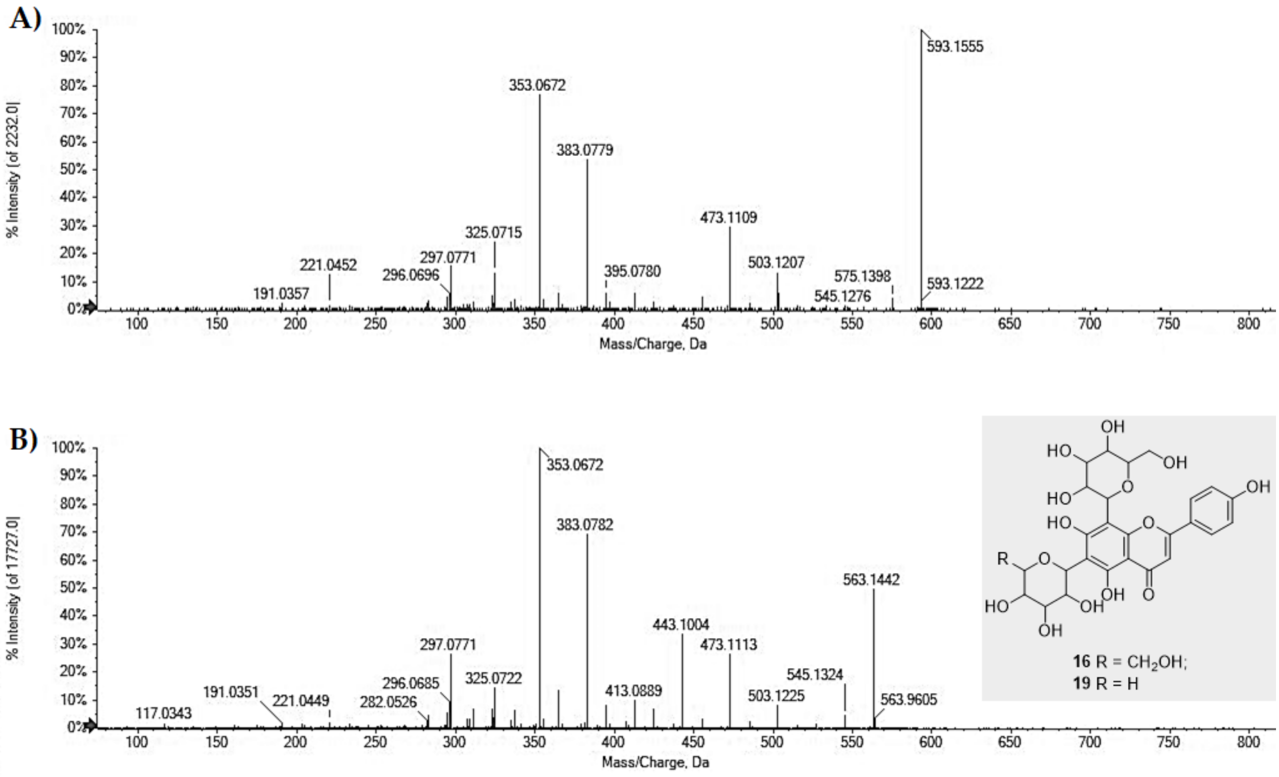

Supplement: Supplementary file 1 [file molecules-25-01765-s001.pdf]
